# Ethanol Reversal of Tolerance to the Respiratory Depressant Effects of Morphine

**DOI:** 10.1038/npp.2015.201

**Published:** 2015-09-09

**Authors:** Rob Hill, Abi Lyndon, Sarah Withey, Joanne Roberts, Yvonne Kershaw, John MacLachlan, Anne Lingford-Hughes, Eamonn Kelly, Chris Bailey, Matthew Hickman, Graeme Henderson

**Affiliations:** 1 School of Physiology and Pharmacology, University of Bristol, Bristol, UK; 2 School of Engineering and Built Environment, Glasgow Caledonian University, Glasgow, UK; 3 School of Clinical Sciences, University of Bristol, Bristol, UK; 4 Division of Brain Sciences, Centre for Neuropsychopharmacology, Imperial College, London, UK; 5 Department of Pharmacy and Pharmacology, University of Bath, Bath, UK; 6 School of Social and Community Medicine, University of Bristol, Bristol, UK

## Abstract

Opioids are the most common drugs associated with unintentional drug overdose. Death results from respiratory depression. Prolonged use of opioids results in the development of tolerance but the degree of tolerance is thought to vary between different effects of the drugs. Many opioid addicts regularly consume alcohol (ethanol), and post-mortem analyses of opioid overdose deaths have revealed an inverse correlation between blood morphine and ethanol levels. In the present study, we determined whether ethanol reduced tolerance to the respiratory depressant effects of opioids. Mice were treated with opioids (morphine, methadone, or buprenorphine) for up to 6 days. Respiration was measured in freely moving animals breathing 5% CO_2_ in air in plethysmograph chambers. Antinociception (analgesia) was measured as the latency to remove the tail from a thermal stimulus. Opioid tolerance was assessed by measuring the response to a challenge dose of morphine (10 mg/kg i.p.). Tolerance developed to the respiratory depressant effect of morphine but at a slower rate than tolerance to its antinociceptive effect. A low dose of ethanol (0.3 mg/kg) alone did not depress respiration but in prolonged morphine-treated animals respiratory depression was observed when ethanol was co-administered with the morphine challenge. Ethanol did not alter the brain levels of morphine. In contrast, in methadone- or buprenorphine-treated animals no respiratory depression was observed when ethanol was co-administered along with the morphine challenge. As heroin is converted to morphine in man, selective reversal of morphine tolerance by ethanol may be a contributory factor in heroin overdose deaths.

## INTRODUCTION

Overdose is the most common cause of accidental death for opiate dependent users, especially if the drugs are injected (Mathers *et al*, 2013; Pierce *et al*, 2015). Death in opioid overdose results primarily from respiratory depression (White and Irvine, 1999). Chronic opioid use results in the development of tolerance such that larger doses are required by an opioid addict to obtain their effects. It has been suggested that tolerance to respiratory depression is less pronounced than to other effects such as euphoria (White and Irvine, 1999). Surprisingly, levels of heroin and its metabolites in overdose victims are often lower than that expected in highly tolerant individuals (Darke *et al*, 2002). This has been interpreted as the victims having taken their normal dose of heroin during a period of reduced tolerance, such as that occurring after incarceration or detoxification, with the drug then inducing greater respiratory depression than expected (White and Irvine, 1999).

Heroin addicts are notorious polydrug users, with alcohol (ethanol), cocaine, benzodiazepines, and amphetamine use reported by heroin users and observed in heroin overdose post-mortem analyses (Darke and Hall, 2003; Hickman *et al*, 2007). Ethanol is the most common drug found along with opioids in overdose victims across several countries, though the levels of ethanol found are rarely high (Darke and Hall, 2003; Hickman *et al*, 2007; Shah *et al*, 2008; Green *et al*, 2011; Fugelstad *et al*, 2014). Furthermore, post-mortem analyses have revealed an inverse relationship between heroin and blood ethanol content (Ruttenber *et al*, 1990; Levine *et al*, 1995; Fugelstad *et al*, 2003).

The prevailing clinical and epidemiological interpretation of these data is that ethanol and heroin mutually re-inforce each other in order to increase the risk of respiratory depression, though other explanations are possible (see Hickman *et al*, 2008). We have focused our research on an alternative hypothesis that ethanol might lower the level of opioid tolerance thus increasing the propensity for overdose. We have reported previously that low doses of ethanol reverse the tolerance induced by prolonged exposure to morphine both at the level of single brain neurons (Llorente *et al*, 2013) and in rodent antinociception tests (Hull *et al*, 2013). In the present study, we have gone on to characterize in mice the development of tolerance to the respiratory depressant effects of three opioids that are important with regard to the abuse and maintenance treatment of opioid addiction—morphine (heroin is rapidly converted to morphine in the body), methadone and buprenorphine—and have examined the ability of low doses of ethanol to reverse such tolerance. We have found that tolerance to the respiratory depressant effect of morphine develops more slowly than the well-documented tolerance that develops to its antinociceptive (analgesic) effect—as hypothesized by White and Irvine (1999). Ethanol reversed morphine-induced tolerance to respiratory depression but not that induced by prolonged administration of methadone. Furthermore, ethanol did not reverse the blockade of morphine-induced respiratory depression produced by prolonged treatment with buprenorphine.

## MATERIALS AND METHODS

### Animals

Male CD-1 mice (Harlan Laboratories, UK) weighing ~30 g were maintained at 22°C on a reversed 12-h dark : light cycle with food and water available *ad libitum*. All experiments were performed in the dark (active) phase. All procedures were performed in accordance with the UK Animals (Scientific Procedures) Act 1986, the European Communities Council Directive 1986 (86/609/EEC) and the University of Bristol ethical review document.

### Measurement of Respiration

Respiration was measured in freely moving animals using plethysmography chambers equipped with differential pressure transducers connected through an interface (emka Technologies, France) to a computer for recording and analysis of respiration parameters. The chambers were supplied with either room air via a pump or cylinder fed a 5% CO_2_ in air gas mixture (BOC Industrial Gases, UK). Rate and depth of respiration were recorded and converted to minute volume. The average minute volume was calculated over 5-min bins.

Mice were habituated to the plethysmography chambers on the day before an experiment. This lasted for 30 min with mice breathing air. On the experimental day, baseline minute volume was measured for all mice breathing 5% CO_2_ in air over a 20-min period. Challenge drugs were injected intraperitoneally (i.p.) in a 5-min window after the baseline measurement and mice returned to the plethysmograph chambers. Minute volume in 5% CO_2_ in air was then recorded for a further 30 min following drug administration.

Changes in minute volume were used to assess respiratory depression following acute drug administration. For each mouse the change in minute volume following acute drug administration was calculated as the percentage of the pre-drug baseline.

### Measurement of Nociception

Mice were hand held, the tails immersed approximately one inch in water at 52.5°C and the latency until the tail was removed from the water measured. On the experimental day, baseline tail-flick latency was recorded prior to drug administration and then measured every 15 min for 1 h. A cutoff time of 20 s was used to prevent thermal damage to the tail.

### Induction of Opioid Tolerance

A 75-mg morphine alkaloid pellet or placebo pellet was implanted subcutaneously on the lower dorsal flank under isoflurane general anesthesia. We found no differences in the responses of placebo pellet-implanted mice and naive mice. Hence, to reduce the number of animals undergoing surgery the control animals in Figure 3c and d and Figure 4b were naive mice rather than placebo pellet-implanted mice. In one series of experiments morphine tolerance was induced by repeated injections with mice receiving two 10 mg/kg morphine injections 12 h apart daily for 5 days. For buprenorphine and methadone, osmotic mini-pumps (ALZET) containing either buprenorphine (6.25 mg/ml delivering 5 mg/kg/day), methadone (75 mg/ml delivering 60 mg/kg/day), or vehicle (saline) were implanted on the dorsal flank under isoflurane general anesthesia. As described previously, to enhance the induction of tolerance to methadone (Quillinan *et al*, 2011) mice received a 5 and 7.5 mg/kg injection of methadone 12 h apart on the day prior to, and an injection of 7.5 mg/kg of methadone on the morning of pump implantation.

### Measurement of Brain and Plasma Morphine Levels

Mice were killed using escalating CO_2_ and blood samples collected from the descending abdominal aorta. Blood samples were centrifuged at 3000 *g* for 10 min at 4°C and the aliquoted plasma supernatant stored at −20°C. Approximately, 100 μl of each plasma supernatant was mixed thoroughly with 500 μl acetonitrile containing 200 ng/ml of deuterated morphine as internal standard and centrifuged at 13 000 r.p.m. for 10 min at room temperature. Approximately, 300 μl of samples of the supernatant were evaporated to dryness using a speed vac.

Immediately after blood sampling, mice were decapitated and the head placed on ice. After removal from the skull, the brains were flash frozen in liquid nitrogen before storage at −80°C. Brains were homogenized in phosphate buffer solution added at a ratio of 2 ml per gram of brain matter. Approximately, 100 μl of aliquots of brain homogenate samples were mixed thoroughly with 500 μl acetonitrile containing 200 ng/ml of deuterated morphine as internal standard and extracted as described for plasma samples.

Brain and plasma samples were reconstituted in acetonitrile/H_2_O (20/80) and analyzed by liquid chromatography (Ultimate 3000 LC system, Dionex, USA)/tandem mass spectrometry (Q Exactive Orbitrap, Thermo Scientific, USA). Samples were analyzed in positive ion mode for morphine, hydromorphone, and morphine-3-glucuronide (M-3-G), the major metabolite of morphine in mice (Kuo *et al*, 1991). The quantification range for morphine was between 2.0 and 20 000 ng/ml. Hydromorphone was not found in any of the samples.

### Measurement of Plasma Corticosterone Levels

Mice were killed by cervical dislocation, decapitated, and trunk blood collected. Approximately, 100 μl of 100 units/ml of heparin were added to each blood sample to prevent coagulation. Samples were then centrifuged at 3000 *g* for 10 min at 4°C, and the supernatant was removed and stored at −20°C prior to analysis. Corticosterone concentrations in the plasma were quantified by radioimmunoassay as previously described (Waite *et al*, 2012) using a corticosterone antibody (supplied by G Makara, Institute of Experimental Medicine, Budapest, Hungary).

### Measurement of Mouse Locomotion

A beam break rig (Linton Instrumentation, UK) was used to assess the locomotor activity of mice. An automated data logging suite (AMON Lite, Linton Instrumentation, UK) was used to track the movement of mice throughout the experimental session. On the day prior to locomotor assessment each mouse was placed in a fresh cage and allowed to explore the cage for 30 min. On the experimental day, the mouse was again allowed to explore the cage for 30 min before drug administration. Locomotion was then measured for 30 min following drug administration. Mice had access to water *ad libitum* but had no access to food in either session in order to dissuade rearing and climbing behavior.

### Data Analysis

Area under the curve (AUC) was determined using a 100% baseline. Overall changes from a single factor (ie, drug) were analyzed using a one-way ANOVA with Bonferroni's post-test. Interaction between prolonged drug treatment (±morphine pellet or osmotic mini-pump) and challenge drug was analyzed using a two-way ANOVA in a two-by-two factorial. Changes in groups over time with repeat measurements were analyzed using a two-way repeated measures ANOVA with Bonferroni's post-test to analyze drug effect over time. GraphPad Prism 4 was used for all statistical analyses. All data are displayed as mean±SEM.

### Drugs and Chemicals

Buprenorphine hydrochloride (Tocris, UK), ethanol (Sigma-Aldrich, UK), methadone hydrochloride (Sigma-Aldrich, UK), and morphine hydrochloride (Macfarlane Smith) were dissolved in sterile saline. 75 mg morphine alkaloid pellets and placebo pellets were obtained from the National Institute on Drug Abuse (Bethesda, MD). Heparin (Sigma-Aldrich, UK) was dissolved in distilled water.

## RESULTS

### Morphine-Induced Respiratory Depression

**Figure 1:**
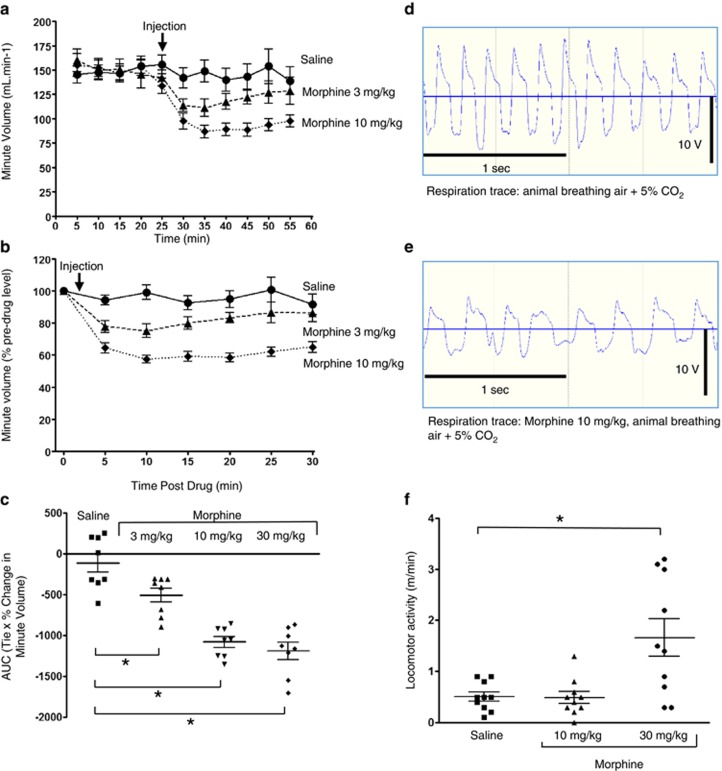
Morphine depression of mouse respiration. (a–c) Morphine (3–30 mg/kg) dose-dependently depressed mouse respiration. In (a) data are presented as minute volume, whereas in (b) the percentage change in minute volume following drug injection for each animal has been normalized to the pre-drug control level before the mean change was calculated. In (c) the area under the curve (AUC) for the percentage change in minute volume has been calculated for each individual animal before the mean AUC has been calculated. *N*=8 for all groups. F=23.17, 3 mg/kg, *p*<0.05; 10–30 mg/kg, *p*<0.001. (d, e) Raw respiration traces recorded from a single mouse in each case. The horizontal blue line indicates the point of pressure inflection. On the respiration traces expiration is upwards. (f) Morphine 30 mg/kg caused a significant increase in locomotor activity, whereas 10 mg/kg morphine did not. *N*=10 for all groups. F=7.83, *p*<0.01. All drugs administered i.p. Data are expressed as mean±SEM and were analyzed using one-way ANOVA with Bonferroni's comparison. *Indicates significant difference (*p*<0.05).

We have studied the effects of morphine on mice breathing 5% CO_2_ in air. In this gas mixture, respiration (tidal volume, not rate) is elevated over that observed when animals breathed air alone (Table 1), but remained at a constant level throughout the period of testing up to 1 h (Figure 1a). Administration of morphine (3–30 mg/kg i.p.) produced significant dose-dependent depression of respiration, which developed rapidly within 5 min of drug injection, and was maintained for the remainder (30 min) of the observation period (Figure 1a–c). The depression of respiration resulted from both a decrease in rate and depth of respiration (compare experimental traces in Figure 1d and e), but there was no decrease in tidal volume (Table 2) as the duration of inspiration was increased. As the experiments were performed in 5% CO_2_ in air, it is not possible to tell if the decrease in minute volume induced by morphine is due to an action on respiratory rate generation or on chemoreflexes. Mice did not exhibit ribcage muscle stiffness, which would reduce tidal volume. As can be seen in Figure 1c, all mice tested responded to morphine with a decrease in respiration, we did not observe any morphine-insensitive animals.

Morphine is known to induce locomotor activity in mice (Lessov and Phillips, 2003; Valjent *et al*, 2010) and as this would likely increase respiration, it could mask the respiratory depressant actions of the drug, especially at high doses. Therefore, in a separate series of experiments, we measured locomotor activity for 30 min after morphine (10–30 mg/kg) injection (Figure 1f). Only at 30 mg/kg did morphine increase locomotor activity. For this reason, we have chosen in our subsequent experiments to use a dose of 10 mg/kg morphine as the challenge dose, as the results would not be confounded by any change in locomotor activity.

One possibility that we wished to exclude was that having the animals breathing 5% CO_2_ in air might induce stress and that the depression of respiration was due to an antianxiety effect of morphine, not a direct effect on respiration. We therefore measured plasma corticosterone levels in animals that had breathed air or 5% CO_2_ in air for 30 min. Plasma corticosterone levels have been shown to rise when animals are stressed (Aliczki *et al*, 2013; Sakakibara *et al*, 2010). There was no difference in the plasma corticosterone levels in animals breathing air or 5% CO_2_ in air (233.7±23.1 and 229.2±34.6 ng/ml, respectively, *N*=10). Furthermore, when animals breathing 5% CO_2_ in air were administered morphine (10 mg/kg), there was no significant decrease in plasma corticosterone levels (177.4±20.7 ng/ml, *N*=10, data were compared using one-way ANOVA with Bonferroni's comparison). These results indicate that morphine depressed respiration directly and not through an indirect anxiolytic mechanism.

### Induction of Morphine Tolerance

**Figure 2:**
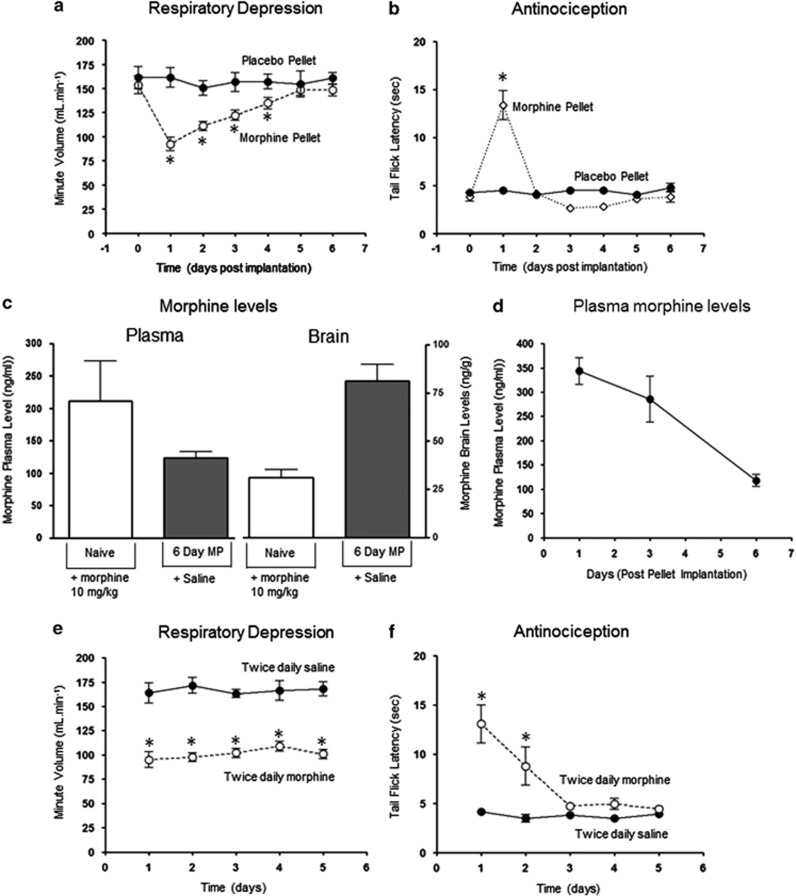
Development of tolerance to the respiratory depressant and antinociceptive effects of morphine. (a) Following implantation of a 75-mg morphine pellet respiratory depression was observed on days 1–4, before returning to baseline levels. F=4.16, *p*<0.001. (b) Following implantation of the morphine pellet antinociception was only observed on day 1. F=26.2, *p*<0.001. In (a) and (b) control animals were implanted with a placebo pellet. (c) Plasma and brain levels of morphine either 15 min after a morphine injection (10 mg/kg i.p.) or 6 days after implantation of a 75-mg morphine pellet. (d) Plasma levels of morphine after implantation of a 75-mg morphine pellet. (e, f) With twice daily acute injections of morphine (10 mg/kg i.p.) no decrease (ie, tolerance) to the respiratory depressant effect of morphine was observed over 5 days but tolerance to the antinociceptive effect of morphine had developed by day 3 (respiratory depression, F=124.7, *p*<0.001; antinociception, F=7.90, *p*<0.001). All drugs administered i.p. except for pellet implantation. Data are expressed as mean±SEM and were analyzed using two-way repeated measures ANOVA with Bonferroni's comparison. *Indicates significant difference (*p*<0.05). *N*=8 for (a–d) and 6 for (e, f).

To induce tolerance to morphine, we exposed animals to morphine continuously for up to 6 days by implanting a morphine pellet (75 mg) subcutaneously. Animals that had been implanted with a morphine pellet showed significant respiratory depression (Figure 2a) as well as antinociception in the tail-flick latency test (Figure 2b) when tested 1 day later, whereas those animals that received a placebo pellet did not. Although the depression of respiration declined on subsequent days, it did not reach pre-pellet levels until 5 days after pellet implantation even though the levels of morphine in the brain and plasma were still elevated (Figure 2c and d). The plasma level of morphine after 6 days of pellet implantation was only slightly lower than that achieved in naive mice following injection with 10 mg/kg morphine (compare data in Figure 2c and d). After 6 days of pellet implantation, mice did not show signs of spontaneous withdrawal such as diarrhea or jumping.

In contrast to respiratory depression, tail-flick latencies returned to baseline 2 days after pellet implantation at a time when plasma morphine levels were still raised (Figure 2d). This suggests that tolerance develops to the antinociceptive effects of morphine more rapidly than to its respiratory depressant effect.

To investigate further the different time courses of tolerance development between the respiratory depressant and antinociceptive effects of morphine, we injected mice twice daily with morphine (10 mg/kg i.p.) for 5 days and measured respiration and tail-flick latency after the second injection on each day (Figure 2e and f). The injection of morphine produced a lower brain level of morphine than morphine pellet implantation (Figure 2c). This protocol produced significant tolerance to morphine-induced antinociception by day 3 (Figure 2f), but tolerance to the respiratory depressant effect of morphine did not develop (Figure 2e). Taken together, these results demonstrate a differential development of tolerance to morphine-induced respiratory depression and antinociception.

**Figure 3:**
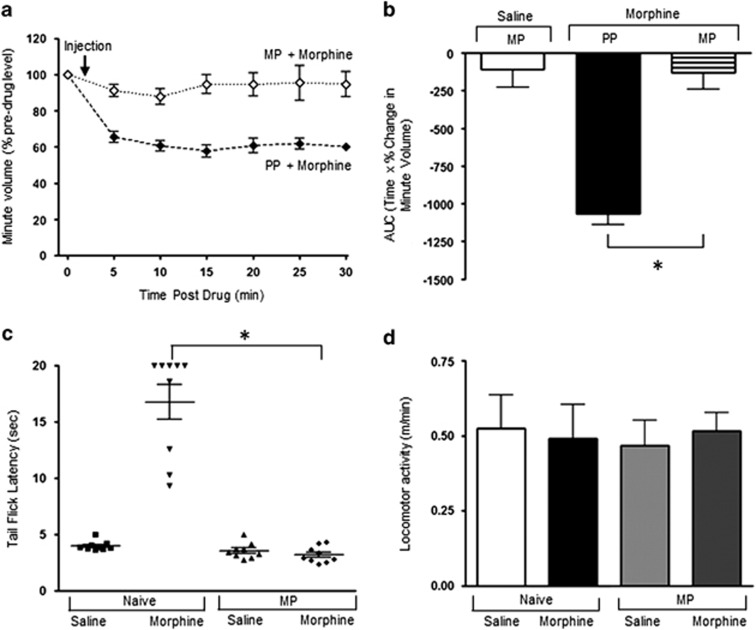
Effect of acute morphine challenge after prolonged treatment with morphine. (a, b) Acute injection of morphine (10 mg/kg) produced significantly less depression of respiration in mice that had been implanted for 6 days with a 75-mg morphine pellet (MP) than in mice implanted with a placebo pellet (PP). Percentage change in minute volume and area under the curve (AUC) have been calculated as described for Figure 1. F=13.13, *p*<0.001. (c) Acute injection of morphine (10 mg/kg) produced significantly less antinociception in mice that had been implanted with a morphine pellet for 6 days. F=68.89, *p*<0.001. (d) Following 6 days of morphine pellet implantation there was no increase in locomotor activity in response to an acute injection of morphine (10 mg/kg). All drugs administered i.p. (other than pellet implantation). Data are expressed as mean±SEM and were analyzed using two-way ANOVA with Bonferroni's comparison. *p<0.05; *N*=6 for all groups.

To facilitate quantification of the level of tolerance to respiratory depression after prolonged (6 days) morphine exposure, we measured the degree of respiratory depression in response to an acute challenge dose of morphine. Mice implanted with a 75-mg morphine pellet for 6 days exhibited a significantly reduced depression of respiration following an acute dose of morphine (10 mg/kg i.p.) on day 6 compared to placebo pellet-implanted mice (Figure 3a and b). Mice implanted with the morphine pellet for 6 days also showed tolerance to the antinociceptive effect of the acute challenge with morphine (Figure 3c). The decreased respiratory depressant effect of morphine after prolonged morphine treatment was not due to the animals becoming sensitized to the locomotor effect of the acute challenge with morphine (10 mg/kg) as locomotor activity remained unchanged (Figure 3d).

### Ethanol Reversal of Morphine Tolerance

**Figure 4:**
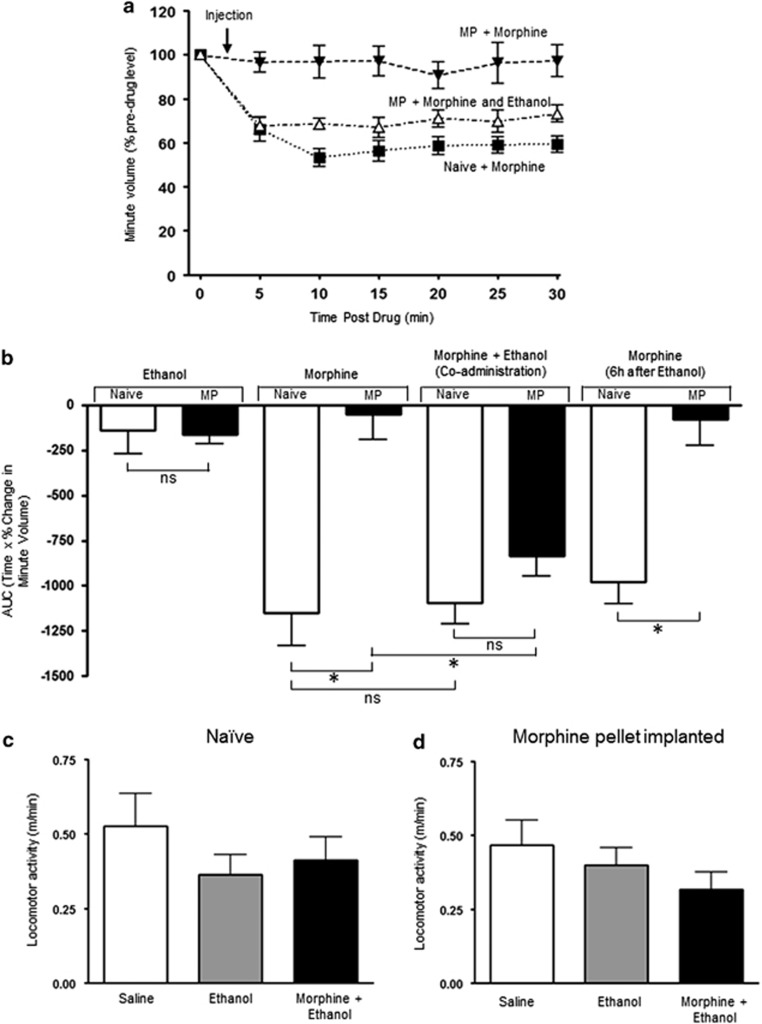
Effect of ethanol on morphine tolerance. (a, b) Administration of ethanol (0.3 g/kg) alone caused no significant depression of respiration in naive mice or mice that had been implanted with a morphine pellet (MP) for 6 days. In 6 days, morphine pellet-implanted mice co-injection of ethanol (0.3 g/kg) and morphine (10 mg/kg) resulted in significant depression of respiration compared to morphine pellet-implanted mice that received only a morphine injection (F=10.82, *p*<0.001), whereas when ethanol (0.3 g/kg) was injected 6 h prior to the morphine challenge no depression of respiration by morphine was observed. (c, d) No significant changes in locomotor activity were observed after any treatment. All drugs administered i.p. (other than pellet implantation). Data are expressed as mean±SEM. In (b), two-way ANOVA with Bonferroni's correction in a 2 × 2 factorial was used; in (c, d) one-way ANOVA with Bonferroni's correction was used. *p<0.05; *N*=6 for all groups except in (b) for morphine 6 h after ethanol where *N*=5.

To investigate the effect of ethanol on morphine tolerance, we gave mice an acute injection of ethanol along with the challenge dose of morphine. We first demonstrated that a low dose of ethanol (0.3 g/kg i.p.) alone did not depress respiration in naive mice or mice that had been implanted with a morphine pellet for 6 days (Figure 4). Furthermore, ethanol (0.3 g/kg i.p.) did not enhance the respiratory depressant effect of the acute morphine (10 mg/kg) challenge (Figure 4b). Higher doses of ethanol (>1 g/kg) did depress respiration in naive mice (data not shown). When mice that had been implanted with a morphine pellet for 6 days received an injection of ethanol (0.3 mg/kg) at the same time as the morphine challenge then morphine now significantly suppressed respiration by reducing respiratory rate (Table 2). This is consistent with an ethanol reversal of morphine tolerance, in that the animals showed significantly greater respiratory depression in response to the acute challenge with morphine (10 mg/kg) than those morphine pellet-implanted mice that received either morphine or ethanol alone (Figure 4a and b). When ethanol (0.3 mg/kg) was administered to morphine pellet-implanted mice 6 h before the morphine challenge then no reversal of tolerance was observed indicating that the reversal of tolerance by ethanol is transient. Ethanol (0.3 g/kg), alone or in combination with morphine (10 mg/kg) did not significantly alter locomotor activity in naive mice or mice that had been implanted with a morphine pellet for 6 days (Figure 4c and d).

### Lack of Effect of Ethanol on Morphine Levels in the Brain and Plasma

**Figure 5:**
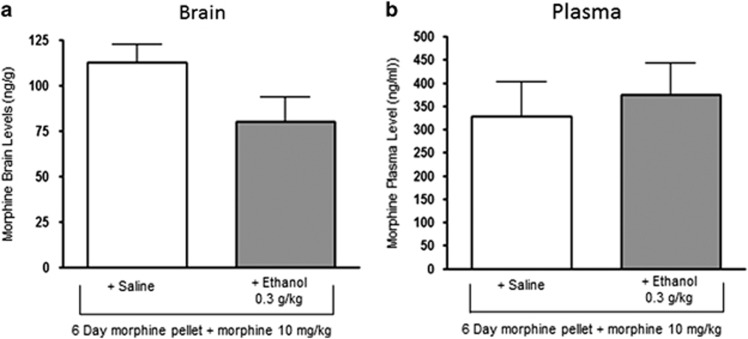
Effect of ethanol on brain and plasma morphine levels. Brain levels (a) and plasma levels (b) of morphine in mice that had been implanted with a 75-mg morphine pellet for 6 days and then injected with either morphine (10 mg/kg)+ethanol (0.3 g/kg) or morphine (10 mg/kg)+saline 15 min before killing the mice. Data are expressed as mean±SEM and were analyzed using Student's unpaired *t*–test. *N*=8 for all groups.

We next determined the concentration of morphine and its major metabolite M-3-G in the brain and plasma of morphine-treated mice (Figure 5). In morphine pellet-implanted animals the plasma level of M-3-G was ~20 times that of morphine, whereas in the brain the concentrations were similar (data not shown). In animals that had been treated with morphine for 6 days, ethanol (0.3 g/kg) administered along with a challenge dose of morphine (10 mg/kg) did not alter the brain concentration of morphine (Figure 5). Thus the enhanced respiratory depressant effect of morphine in morphine-treated mice seen on co-administration of ethanol does not result from ethanol increasing the brain concentration of morphine. This suggests that ethanol reverses morphine tolerance rather than increases morphine levels in the brain.

### Lack of Effect of Ethanol following Chronic Treatment with Methadone or Buprenorphine

**Figure 6:**
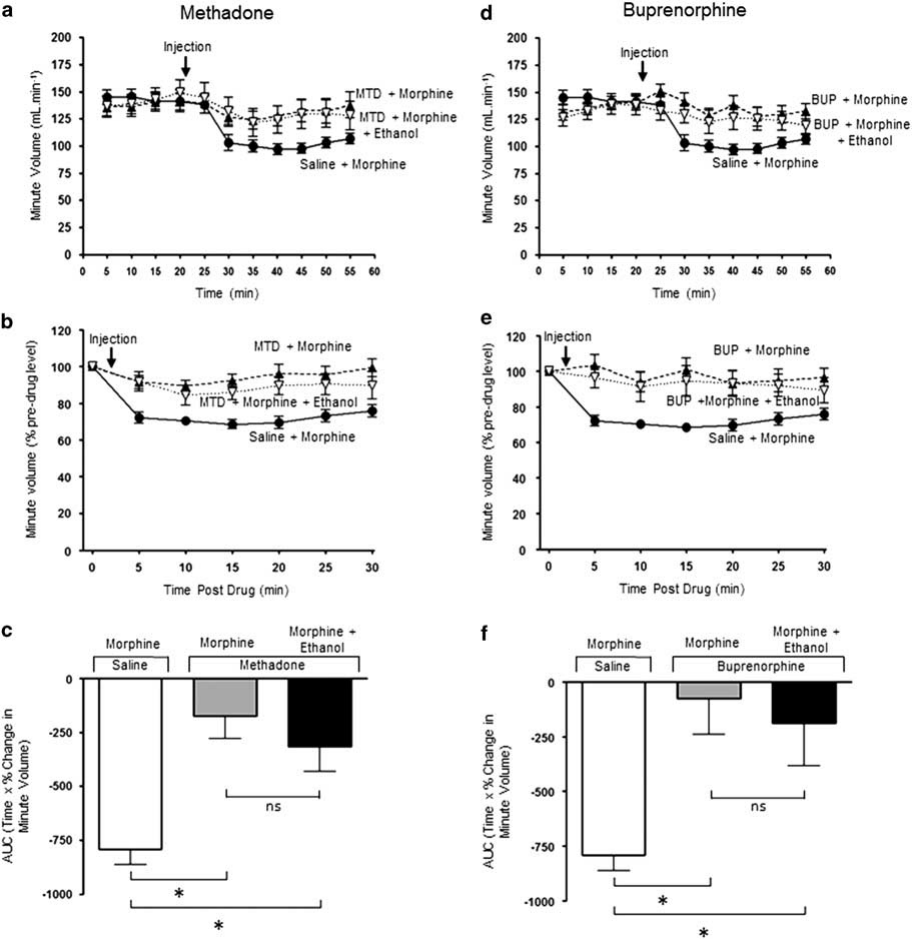
Effect of ethanol on the respiratory depressant effect of morphine after prolonged treatment with methadone or buprenorphine. (a–c) Acute injection of morphine (10 mg/kg) after 6 days of methadone treatment produced significantly less respiratory depression compared to control, non-methadone-treated mice. F=12.31, *p*<0.01. Co-administration of ethanol (0.3 g/kg) with the morphine injection did not increase the amount of respiratory depression. (d–f) Acute injection of morphine 10 mg/kg after 6 days of buprenorphine treatment produced significantly less respiratory depression compared to control, non-buprenorphine-treated mice. F=7.61, *p*<0.01. Co-administration of ethanol 0.3 g/kg with the morphine injection did not increase the amount of respiratory depression. All drugs administered i.p. (other than minipump implantation). Data are expressed as mean±SEM and were analyzed using one-way ANOVA with Bonferroni's comparison. *p<0.05; *N*=12 for all groups that did not receive ethanol. *N*=8 and 9 for methadone and buprenorphine groups, respectively, that received ethanol. The same saline control data are shown in panels (c) and (f) as the saline pump, methadone pump, and buprenorphine pump experiments were carried out in parallel.

After 6 days of methadone or buprenorphine treatment using osmotic mini-pumps (see Materials and Methods), respiration levels were similar to pre-pump implantation levels (compare preinjection levels in Figure 1a and Figure 6a and b). Following 6 days of methadone or buprenorphine treatment, an acute challenge with morphine (10 mg/kg) produced little respiratory depression (Figure 6). For methadone treatment, this likely reflects the development of tolerance but with buprenorphine, which is a *μ*-opioid receptor (MOPr) partial agonist that dissociates slowly from the receptors, we cannot discriminate between buprenorphine's antagonist activity occluding the effect of the morphine challenge and the development of tolerance.

In contrast to what was observed with morphine treatment, in methadone- or buprenorphine-treated animals co-administration of ethanol (0.3 g/kg) with the acute morphine (10 mg/kg) challenge did not result in a significant increase in the ability of morphine to depress respiration (Figure 6a–d).

## DISCUSSION

Morphine-induced respiratory depression, antinociception (analgesia), and reward (a surrogate measure of euphoria in animals) result from activation of MOPr as these behaviors are not observed in the MOPr knockout mouse (Matthes *et al*, 1996; Romberg *et al*, 2003). It has previously been demonstrated that the development of tolerance to the antinociceptive effect of morphine is greater with continuous morphine exposure than with regular intermittent administration (Dighe *et al*, 2009). In the present study, we observed that with twice daily injections of morphine tolerance developed to antinociception but not to respiratory depression, whereas with continuous 6-day morphine administration tolerance developed to both behaviors but the tolerance to antinociception developed faster. McGilliard and Takemori (1978) reported that respiratory depression remained relatively unchanged while tolerance developed to antinociception in mice receiving a combination of morphine injections and pellet implantation but in their study animals were only exposed to morphine for 3 days. Tolerance to respiratory depression was not observed in several other studies in which the duration of continuous morphine administration was short (Ling *et al*, 1989) or doses given once or twice daily (Paronis and Woods, 1997; Kishioka *et al*, 2000).

On prolonged agonist exposure, the MOPr desensitizes and this desensitization contributes to the development of tolerance. The mechanisms responsible for MOPr desensitization are agonist specific; for desensitization induced by morphine, a relatively low-efficacy agonist, there is good evidence for the involvement of protein kinase C (PKC; for extensive review see Williams *et al*, 2013). We and others have provided evidence that cellular tolerance to morphine and tolerance to its antinociceptive effects are mediated by PKC (Inoue and Ueda, 2000; Bohn *et al*, 2002; Smith *et al*, 2002; Bailey *et al*, 2006; Bailey *et al*, 2009a). The specific isoforms of PKC thought to be involved in MOPr desensitization and morphine tolerance are PKC*α*, PKC*γ* and PKC*ɛ* (Smith *et al*, 2007; Bailey *et al*, 2009b). The slower development of tolerance to the respiratory depressant effect of morphine may simply reflect low levels of PKC activity in neurons that control respiration. Expression of constitutively active PKC*α* or PKC*ɛ* in the pre-Bötzinger complex, a group of neurons involved in the generation of respiratory rhythm, increased the development of tolerance to respiratory depression by morphine induced by daily doses of morphine, an effect that afforded increased protection to death by overdose (Lin *et al*, 2012)

The observation that an acute, low dose of ethanol reversed tolerance to the respiratory depressant effects of morphine is in agreement with our previous studies in which we demonstrated that a concentration of ethanol, which would only be mildly intoxicating (20 mM) in man reversed morphine-induced cellular tolerance (Llorente *et al*, 2013), and that low doses of ethanol (0.01–1 g/kg i.p.) similar to that used in the present study (0.3 g/kg i.p.) reversed tolerance to the antinociceptive effects of morphine (Hull *et al*, 2013). It is somewhat surprising that, after 6 days, ethanol injection in morphine pellet-implanted mice did not by itself cause respiratory depression by reversing tolerance to reveal the respiratory depressant effect of the morphine still present in the brain at that time. This may suggest that with long-term exposure a significant amount of the drug is removed from the extracellular space and becomes sequestered in brain tissue such as membrane lipid and therefore is not available for receptor activation.

In brain neurons, ethanol reversal of morphine cellular tolerance was associated with a decrease in MOPr desensitization. Ethanol also reduced MOPr phosphorylation in response to morphine activation of the receptor. This might suggest that ethanol reverses morphine tolerance by directly inhibiting PKC activity but convincing evidence for a direct inhibition of PKC by ethanol has been hard to produce (Slater *et al*, 1997; Rex *et al*, 2008; Reneau *et al*, 2011; Llorente *et al*, 2013). Alternatively, ethanol could decrease MOPr phosphorylation and thus desensitization by increasing phosphatase activity but this seems unlikely, however, as we were unable to observe any effect of ethanol on brain phosphatase activity (Llorente *et al*, 2013), and others have reported that ethanol decreases rather than increases protein phosphatase 2A activity (Hong-Brown *et al*, 2007).

In the present study, prolonged methadone administration induced tolerance to the respiratory depressant effects of morphine—supporting observational evidence and clinical guidance that suggest methadone can provide protection against heroin-related overdose (Cornish *et al*, 2010; Lingford-Hughes *et al*, 2012). Ethanol did not reverse tolerance to morphine induced by methadone. Different opioid agonists desensitize the MOPr by different cellular mechanisms (Kelly *et al*, 2008). Methadone is a high-efficacy MOPr agonist (Rodriguez-Martin *et al*, 2008) that actively recruits arrestin to MOPr (McPherson *et al*, 2010), an effect that requires prior phosphorylation of MOPr by G protein-coupled receptor kinase (GRK) rather than PKC. Failure of ethanol to reverse methadone-induced tolerance would be compatible with the view that it induces tolerance by a different mechanism to morphine.

Buprenorphine binds to MOPr with high affinity and dissociates slowly. It is difficult to displace from the receptor with other ligands, either agonists or antagonists (Lewis, 1985). The reduced response to morphine in buprenorphine-pretreated mice could therefore result from either an antagonist action of buprenorphine or the induction of tolerance by buprenorphine. The experiments we conducted cannot discriminate between these two possibilities. Whichever mechanisms is responsible for the subsequent reduced response to morphine it is not reversed by ethanol.

One complicating factor that we have not yet studied is how the effect on morphine tolerance might change with chronic ethanol consumption. Chronic ethanol exposure has been reported to reduce the coupling of the MOPr to G proteins (Chen and Lawrence, 2000; Sim-Selley *et al*, 2002; Saland *et al*, 2004) and reduce the antinociceptive effect of morphine (He and Whistler, 2011). Thus the effects of acute and chronic ethanol exposure could produce opposite effects on morphine tolerance.

## CONCLUSIONS

Our findings have profound implications for the understanding of opioid-related deaths and the role of alcohol consumption. First, the results explain why the presence of even only moderate amounts of ethanol in the blood can have fatal consequences for opioid addicts who have injected doses of heroin that would not otherwise be expected to lead to overdose, but through reversal of tolerance to morphine-induced respiratory depression rather than acting cumulatively to induce respiratory depression.

We also show that tolerance to the different effects of opioid drugs develops at different rates. As originally hypothesized by White and Irvine (1999) for respiratory depression and euphoria: during dose escalation to maintain responses that undergo rapid tolerance (euphoria), there is an increased likelihood of severe adverse effects (eg, fatal respiratory depression) for responses that undergo little or slowly developing tolerance.

Finally, the fact that ethanol does not appear to reverse tolerance to methadone and buprenorphine highlights the fact that different opioids trigger distinct regulatory mechanisms in the brain (Kelly *et al*, 2008), and also underlines the complexities and dangers of polypharmacy for addicts who may or may not be taking maintenance therapies.

## FUNDING AND DISCLOSURE

The authors declare no potential conflicts of interest.

## Figures and Tables

**Table 1:** Comparison of Respiration Parameters in Mice Breathing Air or 5% CO_2_ in Air

**Gas**	**Minute volume (MV; ml/min)**	**Frequency (F; BPM)**	**Tidal volume (TV; ml/breath)**	***N***
Air	79.2±13.9	414.0±69.8	0.19±0.01	8
5% CO_2_ in air	148.2^a^±7.0	426.9±10.1	0.35^a^±0.01	8

All values are mean±SEM of 5-min averages taken from the 15–20-min time bin following exposure to air or 5% CO_2_ in air in the plethysmograph chamber.

a Indicates significant difference (*p*<0.05) from air. Values were compared using an unpaired Student's *t*-test.

**Table 2:** Effect of Morphine Treatments on Respiratory Frequency and Tidal Volume in Mice Breathing 5% CO_2_ in Air

	**Pre-drug baseline**		**15 Min post-drug**		
**Drug**	**Frequency (BPM)**	**Tidal volume (ml/breath)**	**Frequency (BPM)**	**Tidal volume (ml/breath)**	***N***
Saline	377.4±15.9	0.40±0.02	341.3±18.0	0.43±0.03	8
Morphine 3 mg/kg	416.0±29.6	0.38±0.02	315.0±25.1^a^	0.38±0.02	8
Morphine 10 mg/kg	400.9±21.0	0.37±0.02	265.3±12.1^a^	0.35±0.01	8
Ethanol 0.3 g/kg	426.9±10.1	0.41±0.02	367.5±16.2	0.46±0.02	6
Morphine 10 mg/kg+ethanol 0.3 g/kg	435.9±21.6	0.36±0.04	277.8±11.4^a^	0.35±0.02	6
MP–day 6 +saline	401.6±11.5	0.38±0.01	411.3±16.1	0.39±0.02	8
MP–day 6+morphine 10 mg/kg	395.8±27.0	0.40±0.02	361.0±32.1	0.41±0.03	6
MP–day 6+morphine 10 mg/kg+ethanol 0.3 g/kg	402.6±13.4	0.44±0.03	285.2±8.3^a^	0.43±0.03	6

All values are mean±SEM of 5-min averages. Pre-drug baseline values are taken from the 15–20-min pre-drug time bin. Post-drug values are taken from the 15–20-min time bin taken from the time of injection. Unless otherwise stated there was no significant change from pre-drug baseline levels. MP=75-mg morphine pellet. Values were compared using a paired two-way Student's *t*-test.

a Indicates a significant change (*p*<0.05) from pre-drug baseline values.
